# Antibacterial activity and mechanism of malondialdehyde against *Staphylococcus xylosus* and *Lactiplantibacillus plantarum* isolated from a traditional Chinese dry-cured fish

**DOI:** 10.3389/fmicb.2022.979388

**Published:** 2022-11-01

**Authors:** Qi Zhang, Shiliang Jia, Yicheng Ding, Dongmei Li, Yuting Ding, Xuxia Zhou

**Affiliations:** ^1^College of Food Science and Technology, Zhejiang University of Technology, Hangzhou, China; ^2^Key Laboratory of Marine Fishery Resources Exploitment and Utilization of Zhejiang Province, Hangzhou, China; ^3^National R&D Branch Center for Pelagic Aquatic Products Processing, Hangzhou, China; ^4^Collaborative Innovation Center of Seafood Deep Processing, Dalian Polytechnic University, Dalian, China

**Keywords:** malondialdehyde, antibacterial mechanism, *Staphylococcus xylosus*, *Lactiplantibacillus plantarum*, cellular damage

## Abstract

Malondialdehyde (MDA) is one of the most representative reactive carbonyl species (RCSs) produced by lipid oxidation in food. However, the inhibitory effect of MDA on microorganisms has received little attention. Thus, the aim of this study was to reveal the antibacterial mechanism of MDA on *Staphylococcus xylosus* and *Lactiplantibacillus plantarum* isolated from dry-cured fish. The results showed that the minimum inhibitory concentrations (MICs) of MDA on *S. xylosus* and *L. plantarum* were 90 μg/ml and 180 μg/ml, respectively. Time-kill curves indicated a concentration-dependent antibacterial activity of MDA. Moreover, cell wall damage, cell membrane depolarization, intracellular adenosine triphosphate (ATP) decline, Ca^2+^ and Mg^2+^ leakage, cell morphological destruction and alterations in intracellular biomolecules were observed, which indicated the negative influence of MDA on cell membrane and cellular homeostasis. This study demonstrated the potential antimicrobial properties of MDA and provided theoretical support for the scientific prevention and control of lipid oxidation and microbial contamination in food. This study demonstrated the potential antibacterial properties of MDA and further enriches theoretical studies on the effects of lipid oxidation on microorganisms.

## Introduction

Dry-cured fish is a traditional Chinese food popular among consumers because of its unique flavor and long shelf life. Microbial contamination and lipid oxidation during food manufacture and storage are the key causes of the quality deterioration (Zhang et al., 2021), but the effect of lipid oxidation products on the growth of microorganisms has not been reported.

Numerous studies have been conducted on the antifungal and antimicrobial activity of aldehydes produced by the cleavage of lipid hydroperoxides, including the activity against certain pathogenic strains of these organisms (Anfora et al., 2014). Vaughn and Gardner (1993) showed that four reactive carbonyl species (RCSs) generated by the oxidation of polyunsaturated fatty acids (PUFA) in soybean, including (E)-2-Hexenal, (E)-2-nonenal, (E)-4-Hydroxy-2-nonenal and (Z)-3-nonenal could stress the coexisting fungi. Beltran-Garcia et al. (1997) added volatile aldehydes and ketones extracted from oyster mushroom to bacterial cultures and found that the growth and reproduction of the test strains were completely inhibited. However, the specific coercion mechanisms of aldehydes on the microorganisms remain unclear.

Malondialdehyde (MDA), which is the RCS produced by lipid oxidation in food, has received extensive attention recently. It reacts with myoglobin and myofibrillar proteins, affecting food quality (Schaich et al., 2017). In addition, it can react with proteins and nucleic acids, causing biological damage to cells and carbonyl stress. Therefore, MDA has cytotoxic, neurotoxic, genotoxic and mutagenic properties. However, the effect of MDA on food quality and its antibacterial mechanism have not been paid much attention to.

To the best of our knowledge, current research mainly focused on the antimicrobial mechanisms of biologically active substances, e.g., polysaccharides have been proven to exert their antibacterial action by disrupting cell structure and inhibiting bioenergetic metabolism (Wang et al., 2021). Some polyphenolic actives can inhibit bacterial growth by targeting quorum sensing (QS) systems in addition to bacterial cell membranes, cell walls, proteins, DNA, and other cellular structures (Zheng et al., 2020). It is worth noting that the antibacterial mechanism of fatty acids was studied. It was confirmed that lauric acid inhibited bacterial growth and induced the production of reactive oxygen species through membrane rupture, thereby amplifying the inhibitory effect on sodium taurocholate spores (Yang et al., 2018). Kenny et al. (2009) identified that linoleic acid altered the genes in *S. aureus* that regulate peptidoglycan synthesis on the cell wall. Sun M. et al. (2016) found that docosahexaenoic and eicosapentaenoic acids could completely disrupt the cell membrane of *P. gingivalis*. Additionally, cis-6-hexadecenoic acid could damage membrane integrity by disrupting proton motive force, increasing membrane fluidity and electron transfer pathways (Cartron et al., 2014). However, the stress effect and mechanism of lipid oxidation products in food on microorganisms are rarely studied.

Therefore, the goal of this research was to elucidate the antibacterial effects of MDA on *S. xylosus* and *L. plantarum*. The antibacterial mechanism of MDA was investigated *via* detecting the changes in membrane potential, lactate dehydrogenase (LDH), intracellular ATP, and extracellular Ca^2+^ and Mg^2+^ levels, as well as monitoring the integrity and morphology of cell membrane by microscopic imaging techniques. In addition, the effects of MDA on the biomolecules of *S. xylosus* and *L. plantarum* were investigated by fourier transform infrared spectroscopy (FT-IR). This study will provide a theoretical reference for the effects of lipid oxidation products on microorganisms during aquatic product processing and storage.

## Materials and methods

### Preparation and analysis of MDA

1,1,3,3-Tetraethoxypropane [CAS:122–31-6, HPLC≥96%] was purchased from Sigma-Aldrich (Shanghai) Co., Ltd. Then, 60 μl of 1,1,3,3-Tetraethoxypropane (0.055 g) was diluted to 25 ml with 0.1 M hydrochloric acid in a volumetric flask and hydrolyzed at 40°C for 3 h to prepare an approximately 0.01 M MDA masterbatch. The actual concentration of the MDA masterbatch was determined by measuring the absorbance at 244 nm (*ε* = 13,700). It was stored in a refrigerator at 4°C and kept away from light. The pH was adjusted with 0.1 M NaOH before use and filtered with a 0.22 μm filter.

### Bacteria cultures

The *S. xylosus* B-2, and *L. plantarum* LB-90 used in this study were originally isolated from dry-cured fish samples (*Mylopharyngodon piceus*) by the technical staff in our lab and identified by polymerase chain reaction (PCR) of 16S rRNA. According to Sun Q. et al. (2016), slight modifications were made: *S. xylosus* was maintained on Nutrient Agar (NA) plates at 4°C and *L. plantarum* on de Man Rogosa and Sharp (MRS) agar plates. Thereafter, *S. xylosus and L. plantarum* were incubated in Luria-Bertani (LB) and MRS broth, respectively, for 16–18 h at 37°C until the cells entered the logarithmic growth phase. Next, they were centrifuged at 6000 g for 10 min at 4°C, washed three times with phosphate buffered saline (PBS) and resuspended in PBS at 10^6^–10^7^ CFU/ml for further research.

### Determination of MIC

Minimum inhibitory concentration (MIC) was defined as the very minimal concentration that inhibits the development of the tested bacterium. The determination of MIC was based on the assay proposed by Wu et al. (2022) with some adjustments. Specifically, 100 μl MDA (720, 360, 180, 90, 45, 22.5, 11.25, 5.625 μg/ml) was added to 96-well microplates with LB and MRS broth and 100 μl of bacterial suspension was prepared in accordance with Section “Bacteria cultures,” respectively. The cells were then incubated at 37°C for 12 h and the microbial load was assessed by a microplate reader (Synergy H1, BioTek, Vermont, United States) at 600 nm. Negative controls were filled with 100 μl of bacterial suspension, 100 μl of LB and MRS broth, respectively.

### Growth curves

The procedure of Li et al. was revised to determine the impact of MDA on the growth curve of *S. xylosus* and *L. plantarum* (Liu et al., 2017). In particular, *S. xylosus* and *L. plantarum* were prepared according to Section “Bacteria cultures.” A total of 100 μl of MDA at different concentrations (1/8MIC, 1/4MIC, 1/2MIC, MIC, 2MIC), 100 μl of suspension and the respective broth media were added to 96-well microplates. The cell growth was recorded using a microplate reader (Synergy H1, BioTek, Vermont, United States) at a wavelength of 600 nm after 24 h of incubation at 37°C was recorded. Meanwhile, the negative control group consisted of 100 μl of bacterial suspension and 100 μl of the corresponding LB and MRS broths.

### Lactate dehydrogenase measurement

The determination of LDH was based on Zhang et al. (2018), with some modifications. Bacterial suspensions diluted to 10^6^–10^7^ CFU/ml were centrifuged (4,000 g, 5 min, 4°C) and bacterial precipitates were collected. Except for the control group, *S. xylosus* and *L. plantarum* in the other two groups were treated with MIC and 2 MIC, respectively. Then, the bacteria were incubated at 37°C for 4 h. The supernatant was obtained by centrifuging for 10 min at 6000 g and filtered using a 0.22 μm filter. Next, the supernatant and the appropriate kit (Jiancheng Bioengineering, Nanjing, China) were utilized to measure the concentration of released LDH.

### Determination of the membrane potential

The impact of MDA on the membrane potential in *S. xylosus* and *L. plantarum* was studied using a modified procedure based on the existing approach (Shi et al., 2016). Bacterial pellets of the test strains were prepared according to Section “Bacteria cultures.” After that, cell suspensions were added to 96-well plates containing various doses of MDA (0, MIC and 2 MIC) and incubated at 37°C for 30 min. Next, in each well of a black opaque 96-well microtitration plate, 0.5 μl of 1 mM fluorescent probe, bis-(1,3-dibutylbarbituric acid) trimethynyl oxirane [DiBAC4(3); Molecular probes, (Sigma, Louis, United States)] was added and incubated for 10 min at 37°C in the dark. Afterward, the fluorescence intensity of each well was detected using a multi-mode reader (Synergy H1, BioTek, Vermont, United States) at excitation and emission wavelengths of 492 and 515 nm, respectively, with excitation and emission slit widths of 3 and 5 nm, respectively. The relative fluorescence intensity was used to reflect the change of membrane potential.

### Determination of intracellular ATP

Intracellular ATP was detected using an ATP detection kit (Beyotime Bioengineering Institute, Shanghai, China). Bacterial pellets of *S. xylosus* and *L. plantarum* were prepared based on Section “Bacteria cultures.” With the exception of the control group, the test strains were treated with the MIC and 2MIC levels of MDA. A total of 1 ml of each of these three concentrations was transferred to 1.5 ml centrifuge tubes and incubated at 37°C for 30 min. 100 μl of cell lysis buffer was supplied to each tube for complete cell lysis. The supernatant was acquired by centrifugation (6,000× *g*, 5 min, 4°C) and stored forzen to avoid ATP loss. To eliminate the fluorescent background, 100 μl of ATP assay working solution was poured to each well of a black opaque 96-well microtiter plate for 10 min. The supernatant was then added to the 96-well microplate at a volume of 20 μl. Lastly, a multi-mode reader (Synergy H1, BioTek, Vermont, United States) was used to evaluate background light emission.

### Ca^2+^ and Mg^2+^ measurement

The effect of MDA on Ca^2+^ and Mg^2+^ levels in *S. xylosus* and *L. plantarum* was measured using a modified procedure based on the existing approach (Zhang et al., 2018). The bacterial pellet was collected by centrifugation, and the bacterial suspension was adjusted to 10^6^–10^7^ CFU/ml. Except for the control group, *S. xylosus* and *L. plantarum* in the other two groups were dealed with MDA at MIC and 2MIC levels, respectively, and incubated at 37°C for 4 h. Then, the supernatant was obtained by centrifuging the sample at 4000 g for 10 min and filtering it through a 0.22 m filter. The levels of Ca^2+^ and Mg^2+^ in the sample supernatants were measured at 610 nm and 540 nm using the corresponding kits (Jiancheng Bioengineering, Nanjing, China) and a microplate reader (Synergy H1, BioTek, Vermont, United States), respectively.

### Confocal laser scanning microscopy analysis

The method of Guo et al. (2020) was modified to evaluate the cell membrane damage in *S. xylosus* and *L. plantarum*. The bacterial pellets of *S. xylosus* and *L. plantarum* were prepared based on Section “Bacteria cultures.” With the exception of the control group, the test strains were treated with MDA at MIC and 2MIC levels, respectively. After a 2-h incubation period at 37°C, the sample were washed three times with saline. After that, 3 μl of Propidium iodide (PI)/SYTO9 mixed dye was added and incubated at 37°C in the dark for 15 min. Finally, 10 μl of the cell suspension was placed on a glass slide and fluorescent images were immediately acquired by CLSM (Lsm880, Carl Zeiss, Yarra, Germany).

### Field emission gun scanning electron microscopy analysis

The method of Zhou et al. (2019) was modified to observe the morphology of *S. xylosus* and *L. plantarum* by FEGSEM. The bacterial pellets of *S. xylosus* and *L. plantarum* were prepared as indicated by Section “Bacteria cultures.” *S. xylosus* and *L. plantarum* in the other two groups were dealed with MDA at MIC and 2MIC levels, respectively, aside from the untreated group. Afterwards, samples were incubated at 37°C for 4 h. After centrifugation (6,000× *g* for 10 min at 4°C), the bacterial precipitates were washed three times with PBS (pH 7.0) and homogenized in 2.5% glutaraldehyde in PBS. Then, they were fixed at 4°C for 12 h. The bacterial precipitates were washed twice with PBS and dehydrated in various concentrations of ethanol (30, 50, 70, 80, 90 and 100%) for 10 min each step and then freeze-dried *in vacuo* for 48 h. Subsequently, the lyophilized samples were fixed on a carrier table using a conductive adhesive. At last, the cell morphology was evaluated utilizing FEGSEM (GeminiSEM500, ZEISS, Oberkochen, Germany) after a thin layer of gold was applied to the samples.

### Fourier transform infrared spectroscopy analysis

Bacterial pellets of the test bacteria were prepared according to Section “Bacteria cultures.” The test strain was treated with 2 MIC of MDA and the samples were incubated at 37°C for 4 h, and the control groups were treated with PBS under the same conditions. Bacterial precipitates were then obtained by centrifugation (6,000× *g*, 10 min, 4°C) and rinsed with PBS buffer. To obtain the cell lyophilized powder, the cells were rapidly frozen in liquid nitrogen, crushed to a fine powder, and pre-frozen at −40°C for 24 h, followed by vacuum freeze-drying for 24–48 h. The potassium bromide disc method was used to prepare samples prior to analysis. Three duplicates of each treatment were used. Each treatment group included three replicates.

All spectra were recorded in reflection mode utilizing a FT-IR spectrometer (Bruker Vertex 70, Bruker Optics, Karlsruhe, Germany) and data was collected using Bruker OPUS software (version 7.0; Bruker Optics, Karlsruhe, Germany; Wang et al., 2018). Unscrambler × 10.4 software were used to conduct spectral pretreatment and data analysis.

### Statistical analysis

All of the trials carried out in triplicate. The data was entered into SPSS software (version 22.0; SPSS, Inc., Chicago, United States) for data analysis, and *p* values were generated to illustrate significance, with *p* < 0.05 indicating a significant difference. The mean ± standard deviation (*n* = 3) was used to express all of the data.

## Results

### MICs of MDA against *Staphylococcus xylosus* and *Lactiplantibacillus plantarum*

The MICs of MDA against *S. xylosus* and *L. plantarum* were 90 μg/ml and 180 μg/ml, respectively, which means *S. xylosus* was more susceptible to MDA than *L. plantarum*.

### Effects of MDA on the growth curve of *Staphylococcus xylosus* and *Lactiplantibacillus plantarum*

**Figure 1:**
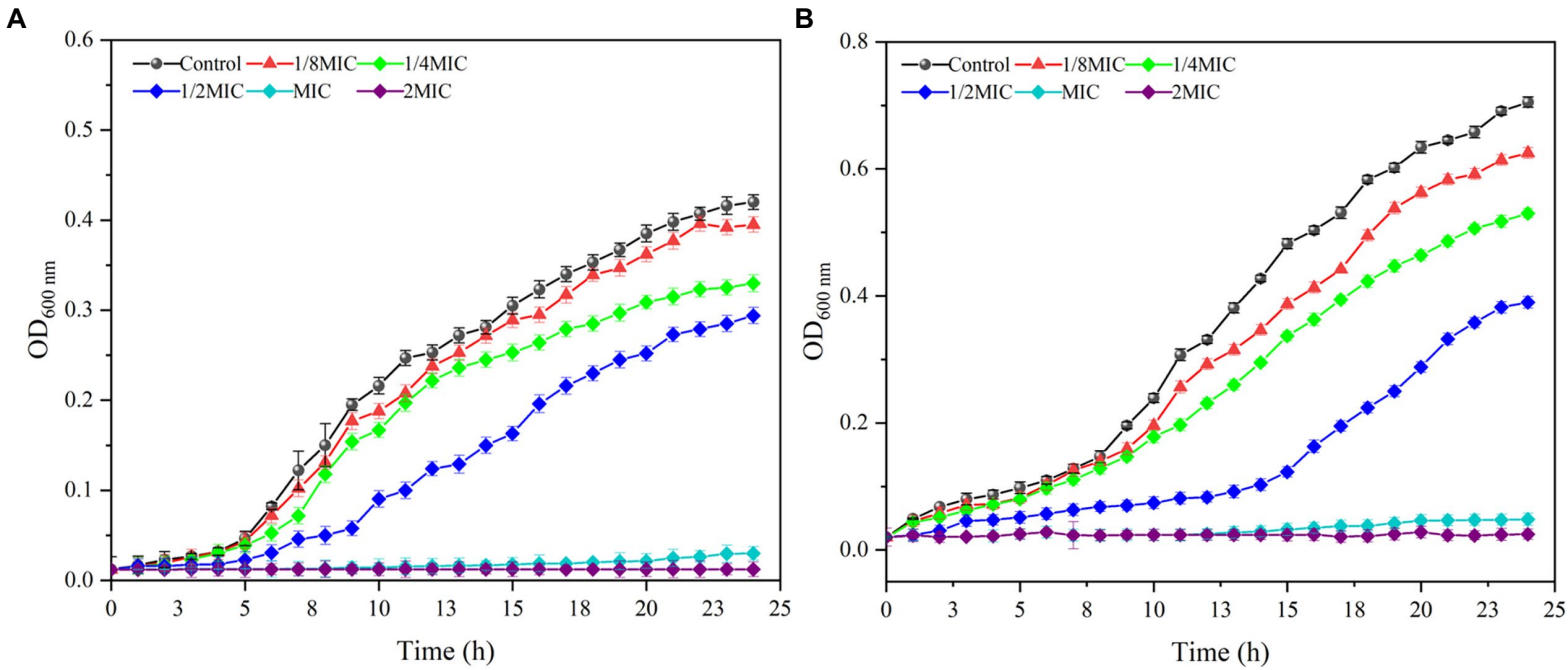
Effects of MDA on the cell growth of *S. xylosus* **(A)** and *L. plantarum* **(B)**.

The inhibitory effects of MDA on the growth of *S. xylosus* and *L. plantarum* were further explored by measuring their OD_600_ at 1 h intervals. As shown in Figure 1, the inhibitory effects of MDA on *S. xylosus* and *L. plantarum* were to prolong their growth delay and reduce their maximum growth rate. At MIC and 2 MIC levels, MDA completely inhibited the growth of *S. xylosus* and *L. plantarum*. Furthermore, at MDA concentration of 1/8 MIC, the effect of MDA on the growth curve of *S. xylosus* within 24 h was not significant. At MDA concentrations of 1/8, 1/4, 1/2 MIC, the growth of *L. plantarum* was initially represses, and such a tendency lasted for 10–16 h, indicating the occurrence of partial bacteria inhibition or lysis by MDA. Moreover, from the overall trendencies of growth curves, it can be concluded that the antimicrobial activity of MDA against *S. xylosus* and *L. plantarum* was dose-and time-dependent.

### Effects of MDA on LDH of *Staphylococcus xylosus* and *Lactiplantibacillus plantarum*

**Figure 2:**
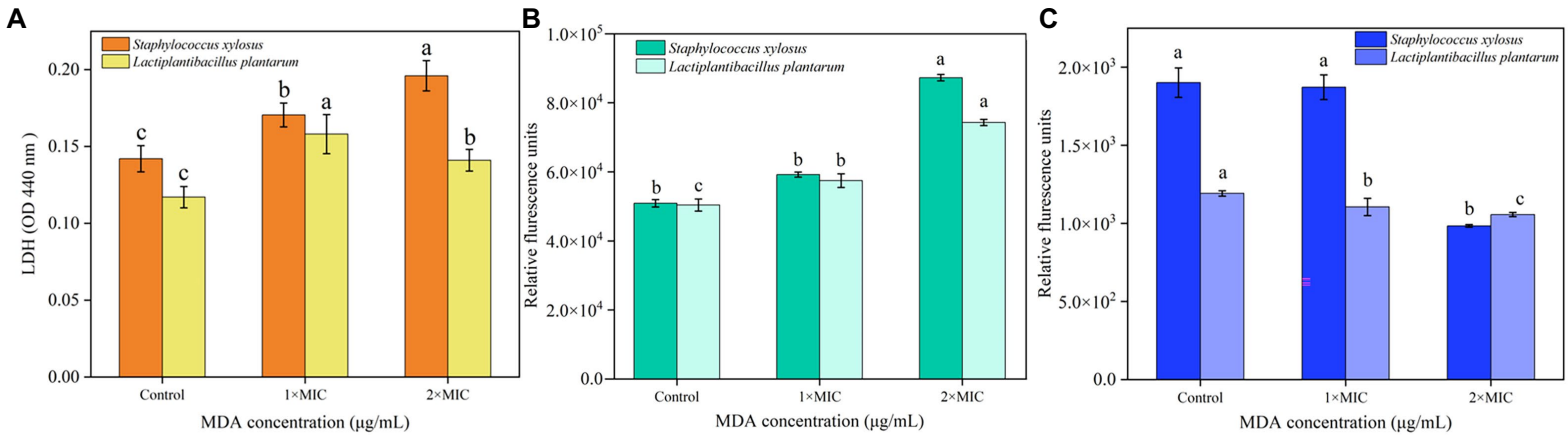
Effects of MDA on **(A)** the changes of LDH release; **(B)** membrane potential; **(C)** intracellular ATP of *S. xylosus* and *L. plantarum.*

The results of the LDH release assay are presented in Figure 2A. The levels of LDH released in the MIC and 2 MIC treated groups of both *S. xylosus* and *L. plantarum* showed an increasing trend compared with the untreated group. It indicated that the wall structure of both *S. xylosus* and *L. plantarum* were damaged after MDA treatment. It is worth noting that the release amount of LDH in the 2MIC-treated group of *L. plantarum* was smaller than that in the MIC group.

### Effects of MDA on the membrane potential of *Staphylococcus xylosus* and *Lactiplantibacillus plantarum*

As shown in Figure 2B, the cell membrane potentials of *S. xylosus* and *L. plantarum* showed a significant difference (*p* < 0.05) after 2 MIC of MDA treatment compared to that of the control. In addition, the depolarization of membrane potential of the *L. plantarum* cell caused by MDA treatment at MIC was significant (*p* < 0.05). Overall, the depolarization of bacterial cell membrane potential caused by MDA treatment showed mass concentration dependence.

### Effect of MDA on intracellular ATP concentration of *Staphylococcus xylosus* and *Lactiplantibacillus plantarum*

The result showed the concentrations of the two strains were significantly reduced (*p* < 0.05) after MDA treatment (2MIC) compared to those of the control group. Whereas no significant changes were found in *S. xylosus* treated with MDA (MIC) compared to the untreated group (*p* > 0.05), *L. plantarum* changed significantly (*p* < 0.05) with the same treatment (Figure 2C). This difference might be due to the difference in the rate of intracellular ATP consumption and synthesis between strains.

### Effects of MDA on Ca^2+^ and Mg^2+^ leakage of *Staphylococcus xylosus* and *Lactiplantibacillus plantarum*

**Figure 3:**
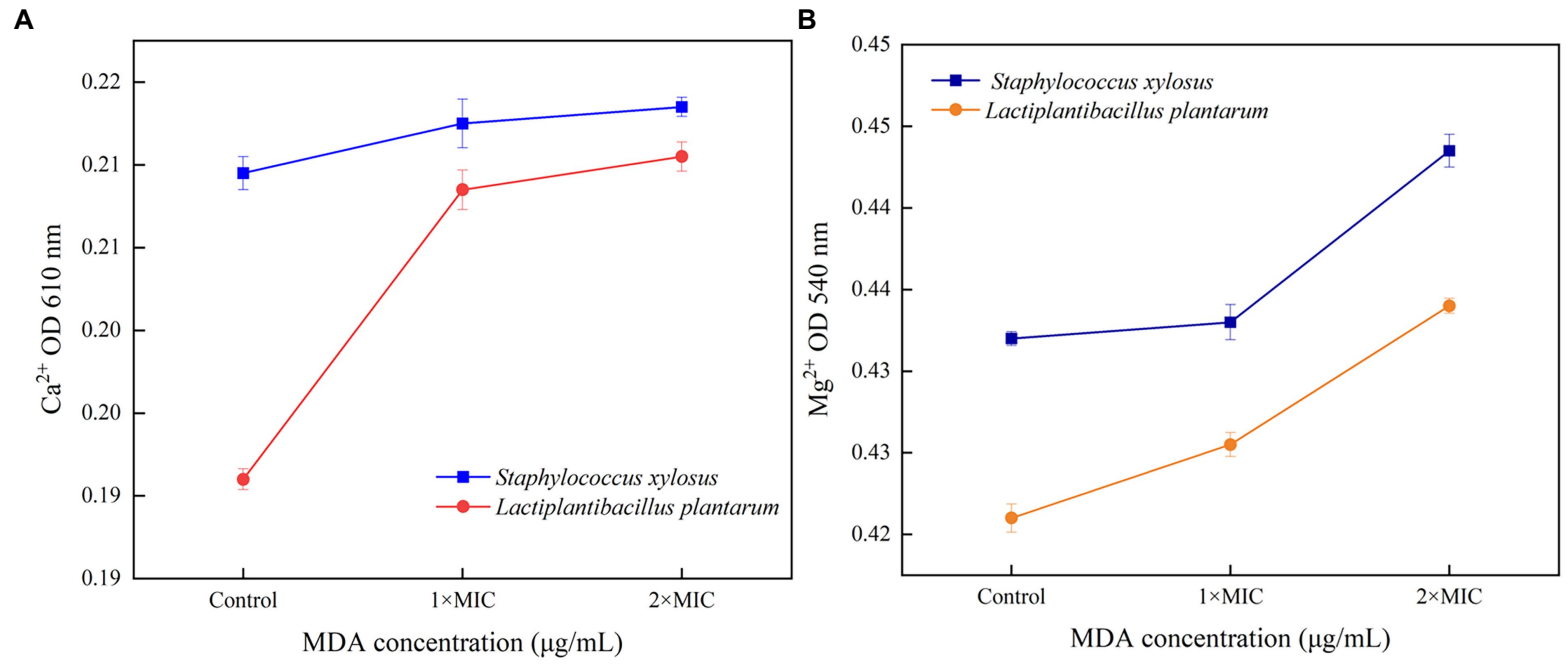
Release of Ca^2+^ **(A)** or Mg^2+^ **(B)** from *S. xylosus* and *L. plantarum* treated with MDA.

As shown in Figure 3, the release of Ca^2+^ and Mg^2+^ in *S. xylosus* and *L. plantarum* increased with MDA concentration. As the amount of MDA increased from MIC to 2 MIC, the release of Ca^2+^ appeared to decrease. The Mg^2+^ release pattern became more obvious with the MDA treatment of 2MIC.

### Effects of MDA on cell membrane damage of *Staphylococcus xylosus* and *Lactiplantibacillus plantarum*

**Figure 4:**
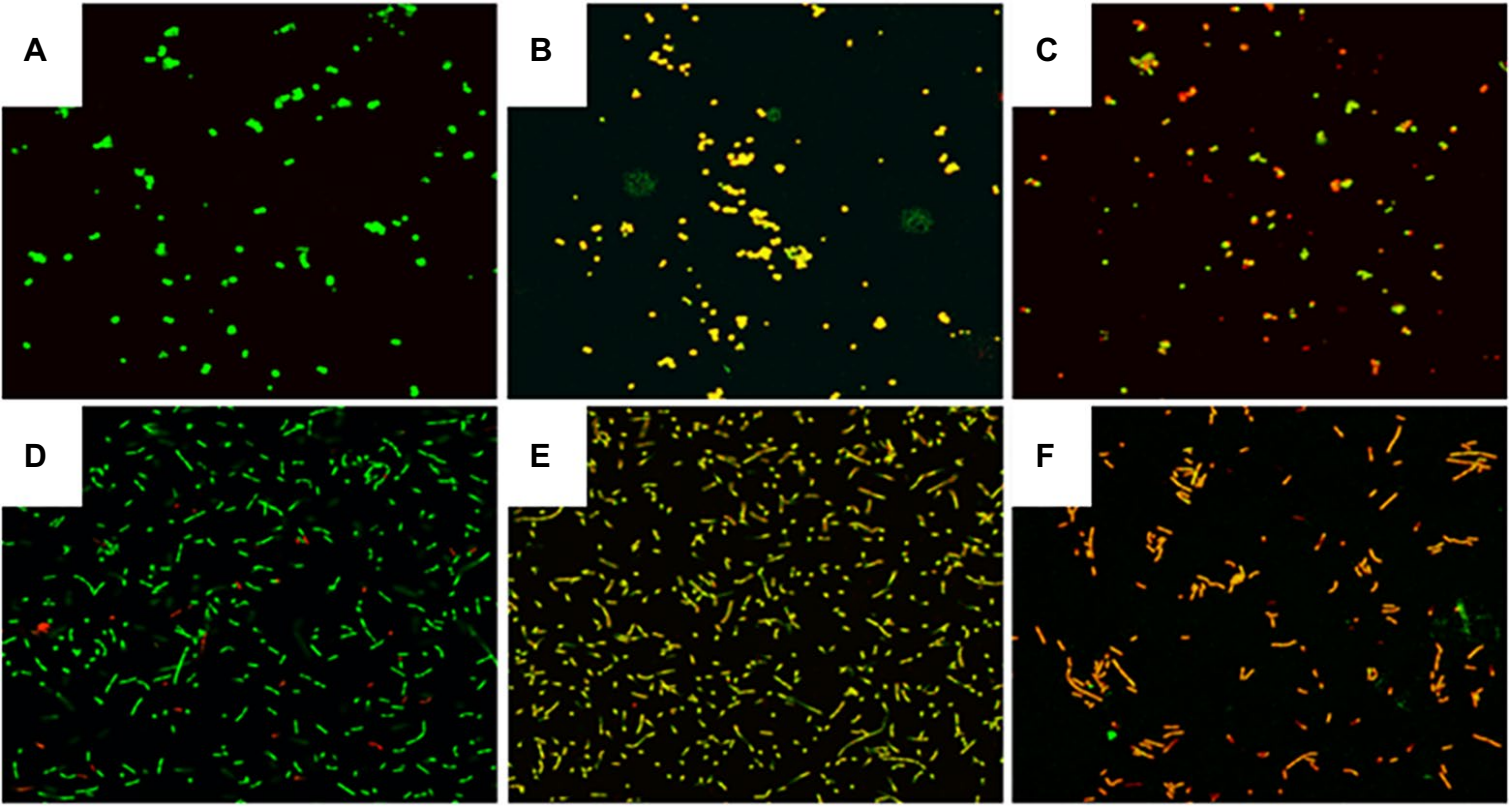
Effect of MDA on the integrity of the cell membrane of *S. xylosus* and *L. plantarum*. **(A)** *S. xylosus* untreated; **(B)** *S. xylosus* treated with MDA at MIC; **(C)** *S. xylosus* with MDA at 2MIC; **(D)** *L. plantarum* untreated; **(E)** *L. plantarum* treated with MDA at MIC; **(F)** *L. plantarum* with MDA at 2MIC.

The cells in control groups fluoresced brightly green, suggesting that the cell membrane was undamaged (Figures 4A,D). In contrast, MDA treatment at MIC, significantly decreased green fluorescence and increased red fluorescence, suggesting that MDA induced cell membrane damage in *S. xylosus* and *L. plantarum* (Figures 4B,E). Fewer cells emitted green fluorescence when cells were treated with MDA at 2MIC (Figures 4C,F), indicating that severe membrane damage had a major impact on cell development and differentiation.

### Effect of MDA on cell morphology of *Staphylococcus xylosus* and *Lactiplantibacillus plantarum*

**Figure 5:**
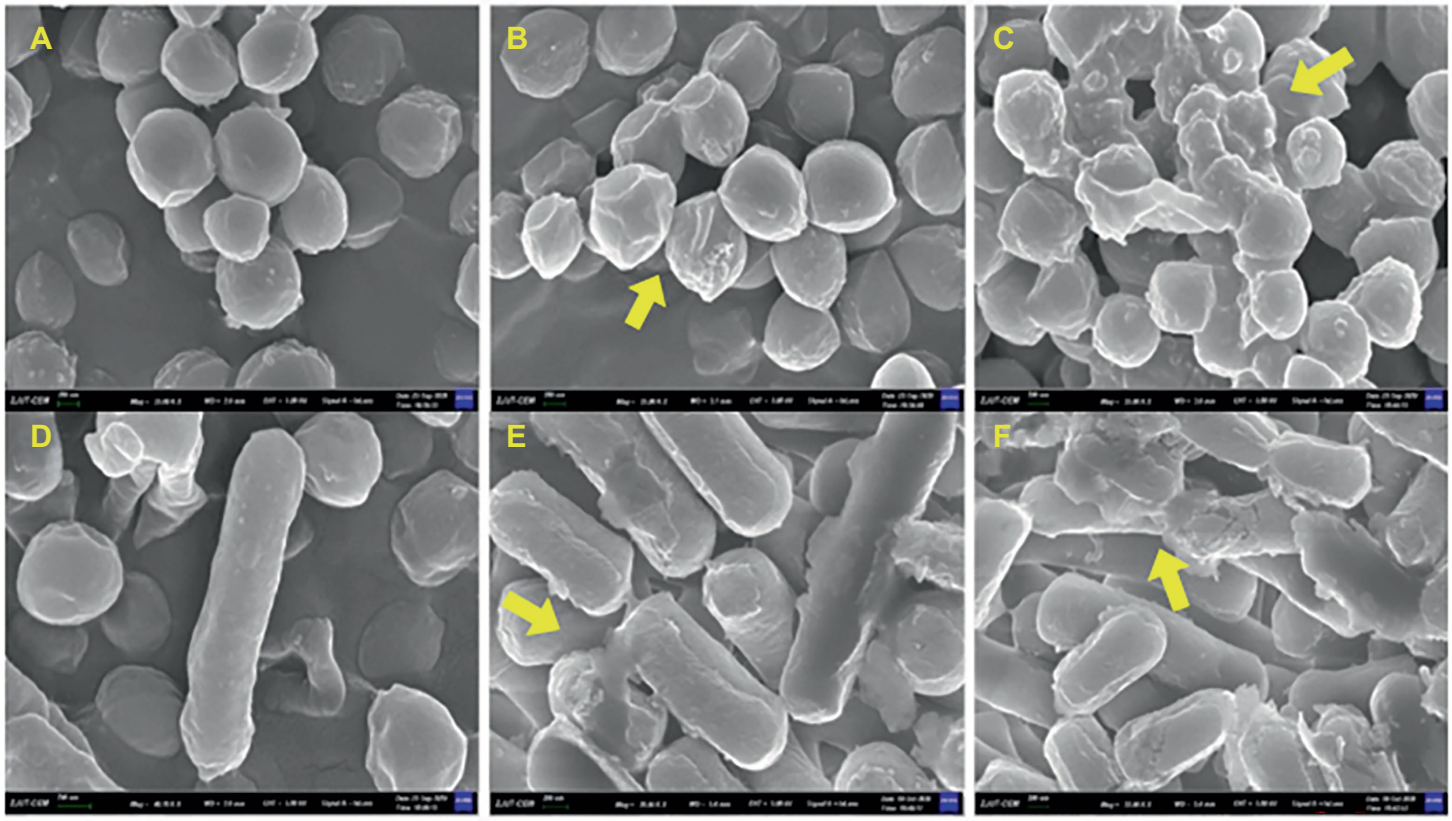
FEGSEM image of *S. xylosus* and *L. plantarum*. **(A)** *S. xylosus* untreated; **(B)** *S. xylosus* treated with MDA at MIC; **(C)** *S. xylosus* with MDA at 2MIC; **(D)** *L. plantarum* untreated; **(E)** *L. plantarum* treated with MDA at MIC; **(F)** *L. plantarum* with MDA at 2MIC.

The effects of MDA on the cell morphology of *S. xylosus and L. plantarum* were observed using field emission scanning electron microscopy. As shown in Figure 5, the untreated bacteria had a regular morphology with a smooth surface. After MDA treatment with MIC, the strains had irregular, wilted and rough surfaces but maintained their original shape (Figures 5B,E). In contrast, MDA treatment at 2MIC significantly depressed the bacterial cell surface, severely disrupted membrane, caused cell damage or cell debris adhesion and aggregation (Figures 5C,F). Furthermore, the degree of membrane injury increased with increasing dosage.

### Effects of MDA on biomolecules in *Staphylococcus xylosus* and *Lactiplantibacillus plantarum*

**Figure 6:**
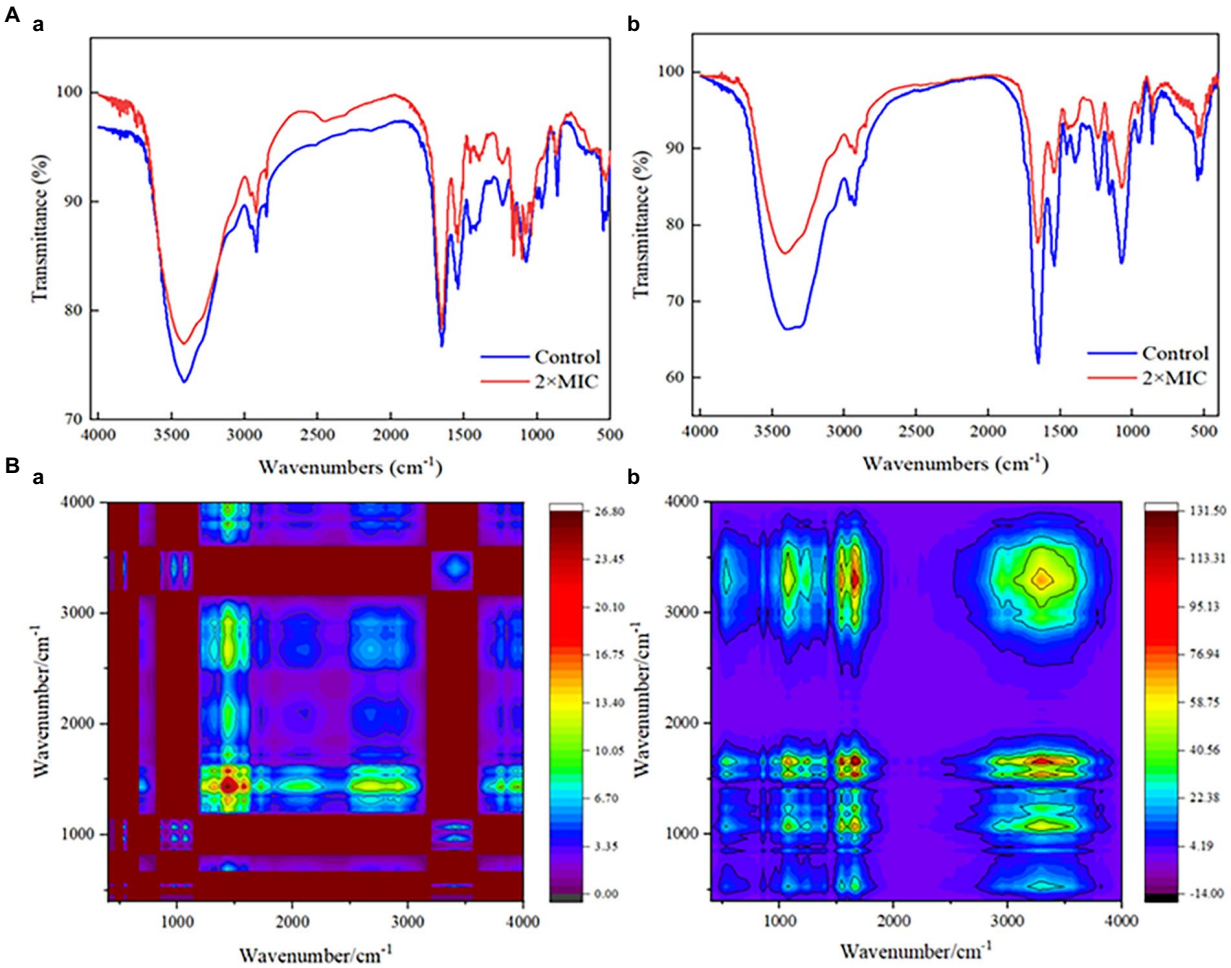
FT-IR spectra analysis of *S. xylosus* and *L. plantarum* under MDA treatment. **A(a)**: IR spectra of *S. xylosus*; **A(b)**: IR spectra of *L. plantarum*; **B(a)**: 2DCOS-IR of *S. xylosus*; **B(b)**: 2DCOS-IR of *L. plantarum*.

The injury mechanisms of MDA to *S. xylosus* and *L. plantarum* were investigated by comparing the FT-IR spectral characteristics of strains in the untreated group and those in the MDA-treated group with 2MIC (Figure 6A). Significant deviations were observed in the spectral intensities and their waveforms in the regions I (3,000–2,800 cm^−1^) and 1,100–900 cm^−1^. In addition, the tension of the 1,600–1700 cm^−1^ (amide I) and 1,520–1,550 cm^−1^ (amide II) peaks of *L. plantarum* was significantly reduced by the action of MDA. The intensity of the protein/amide I and II (1,700–1,500 cm^−1^) and carbohydrate bands (1,100–1,000 cm^−1^) also showed a decrease. Considering that the spectral peaks of the infrared spectrum are seriously overlapped and difficult to resolve, the original spectrum was extended in a second dimension to obtain a two-dimensional correlation spectrum (2DCOS-IR; Figure 6B). This allows the analysis to be significantly enriched and refined, identifying key sites of change in response to external stimuli. Based on the shade of color, most of the spectral differences appeared in the 1,500–1,200 cm^−1^ spectral region of *S. xylosus*, and between 1,700 and 1,500 cm^−1^ of *L. plantarum*.

## Discussion

As a representative product of lipid oxidation, the antibacterial activity of MDA against *S. xylosus in vitro* was higher than that of *L. plantarum*. Time-kill curves showed concentration-dependent activity. In addition, Li et al. (2014) demonstrated that the MIC of 3-poly-lysine against *S. aureus* was lower than that of *E. coli*. Coronel-Leon et al. (2016) found that LAE® inhibits *L. plantarum* at a concentration of 32 μg/ml. For *S. aureus*, the MIC of lactobionic acid is 18.75 mg/ml and the MIC of both shikimic acid and quinic acid is 2.5 mg/ml (Bai et al., 2022). There is no consensus on the standard method for measuring MIC. The purity of the compounds studied, the differences in strains and bacterial cell structures, etc., can lead to variability in measurement.

The cell wall keeps the intracellular substances from leaking out. Between the cell wall and the cell membrane, LDH is an essential glycolytic enzyme (Zhang et al., 2018). When cellular structural integrity is preserved, LDH activity cannot be evaluated in culture medium. Therefore, cell wall injury can be indicated by changes in LDH activity in the culture. In this research, the cell wall structure of both *S. xylosus* and *L. plantarum* was damaged after MDA treatment. However, the release of LDH in *L. plantarum* treated with 2MIC was smaller than that in the MIC group, which was attributed to the release of protease from the cells that hydrolyse LDH, leading to a downward trend. Exposure to unsteady environment over long periods may also result in enzyme deactivation (Zhang et al., 2018). Zhang et al. (2018) also confirmed that LDH activity decreases with the increase of drug concentration and treatment time.

The maintenance of membrane potential indicates membrane integrity, and depolarization of membrane potential means membrane damage. DiBAC4(3) is a lipophilic anionic fluorescent dye that is sensitive to cell membrane potential and is commonly used as a cell membrane potential indicator dye. When the cell is depolarized, the fluorescence emitted by the dye entering the cell is enhanced due to bonding activation (Nuding et al., 2006), and thus the fluorescence value is positively correlated with the degree of membrane damage. This study confirmed that bacterial membrane damage caused by MDA is concentration dependent. Tian et al. (2021) indicated that thymol induced depolarization of *E. sakazakii* bacterial cell membrane, which is caused by the decrease in membrane potential. Another study reported that daptomycin caused depolarization of the membrane potential of *S. aureus*, leading to bacterial cell death (Silverman et al., 2003). Bot and Prodan (2009) suggested that depolarization of the cell membrane potential was caused by the changes in pH or in cell membrane permeability. Therefore, it is hypothesized that MDA on alters the permeability of cell membranes, which in turn changes the cell membrane potential, and inhibits *S. xylosus* and *L. plantarum*.

ATP is a vital energy molecule for bacteria and is involved in a variety of physiological processes. Intact cells have steady ATP levels. However, bacteriostatic substances can disrupt cellular homeostasis and integrity, thereby affecting intracellular ATP concentration and thus exerting bacteriostatic effects (Han et al., 2020). The ATP bioluminescence technique, which utilizes the concept of firefly luciferase reacting with its ATP substrate to catalyze luciferin to produce fluorescence, is used to assess intracellular ATP (Tian et al., 2021). The fluorescence intensity is related to the measured ATP levels. Therefore, the impact of MDA on intracellular ATP concentrations of *S. xylosus* and *L. plantarum* was studied by quantifying the fluorescence intensity.

It was shown that MDA treatment significantly reduced the ATP concentration in the bacteria. The drop in intracellular ATP content might be owing to an increase in the ATP hydrolysis rate caused by the proton pump, resulting in fast ATP depletion or changes in cell membrane permeability, allowing intracellular ATP to leak through the cell membrane (Mempin et al., 2013). Tian et al. (2021) also confirmed that the reduced intracellular ATP in *E. sakazakii* treated with thymol might be due to changes in cell membrane permeability or an increase in ATP hydrolysis rate, resulting in the quick consumption of ATP leakage. Therefore, the decrease in intracellular ATP concentrations of *S. xylosus* and *L. plantarum* in this study suggested that MDA may inhibit their growth by affecting the metabolic functions of cells and bacteria or cell membrane permeability.

Ca^2+^ and Mg^2+^ are important messengers regulating the functions of many cells (Zeng et al., 2021). Among them, Ca^2+^ is required for the maintenance of cytoskeletal structure, cell motility, cell division, intracellular movement and contraction (Visudtiphole et al., 2017). Over 300 metabolic reactions require the participation of Mg^2+^. In addition, Mg^2+^ is essential for the activation of all phosphatases and kinases. This messenger balances the physiological structure of DNA and RNA (Wang et al., 2022). Ca^2+^ and Mg^2+^ also play important roles in maintaining electrolyte and osmolarity balance. The interaction between MDA and membrane structure may be responsible for the rising Ca^2+^ curve. That indicated the cell membrane was damaged, and MDA could affect the metabolic reactions of bacteria. In addition, the decrease in Ca^2+^ release may be due to the recombination of free Ca^2+^ and Ca^2+^-binding proteins (Figure 3A; Zhang et al., 2018). This reorganization was because the unordered cell film structure of bacteria vanished when MDA went through the layer and entered the cell. Subjected to MDA stress, a part of Mg^2+^ may be involved in protein phosphorylation, which results in the production of ATP or other substances to defend or restore the cell from the initial damage. The increase in Mg^2+^ release suggests that it may originate from bacterial death. Overall, the emission of Ca^2+^ and Mg^2+^ demonstrated that MDA enhanced the permeability of *S. xylosus* and *L. plantarum* cell membrane and further affected bacterial metabolism.

The LIVE/DEAD® BacLight™ kit is commonly used to assess membrane integrity (Leuko et al., 2004). Two dyes, fluorescent SYTO 9 and propidium iodide (PI), are included in the package. SYTO 9 may readily pass through any cell membrane, stain nucleic acids, and exhibit green fluorescence. The waterproof red fluorescent PI penetrates the cell through the damaged cell membrane, and is stained by nucleic acids (Boulos et al., 1999). This study confirmed that MDA could induce cell membrane damage in *S. xylosus* and *L. plantarum* by assessing membrane integrity (Figures 4B,E). Similarly, Diao et al. (2014) found that at a mass concentration of 2MIC, fennel essential oil disrupted the cell membrane integrity of *Shigella* spp. Moreover, Shi et al. (2016) found that citral at a mass concentration of 2MIC (1.08 mg/ml) significantly reduced the percentage of bacteria with intact cell membranes in *C. sakazakii* to 15% of the control (*p* < 0.05).

The integrity of the cell membrane is essential for the normal growth and metabolism of bacteria. Disruption of cell membrane integrity may cause the efflux of some important cellular components such as proteins, nucleic acids and sugars, thus affecting the normal growth and metabolism of bacteria (Wu et al., 2022). The cytomorphological observations showed that MDA treatment damaged both *S. xylosus* and *L. plantarum*. Similarly, Li et al. (2014) observed that 3-poly-lysine caused *S. aureus* to lose its rounded, spherical morphology and caused *E. coli* cells to sink and ruffle the cytosol surface, without lysis and rupture phenomena.

The action mechanism of bactericidal substances has recently been studied using FT-IR spectroscopy. It displays and recognizes changes in the metabolomic response of bacterial cellular components. These components include cell membrane fatty acids, proteins/pep-peptides and cell wall polysaccharides or carbohydrates. The effectiveness of FT-IR spectroscopy in examining the overall molecular composition of microbial cells in reaction to external influences is well established, and it has grown in popularity in microbiological research. As shown in Supplementary Table S1, the FT-IR spectra of the bacterial might be divided into five regions (Tantala et al., 2019). Significant differences in the spectral region of the region I (3,000–2,800 cm^−1^) indicated alterations in cell membrane lipids, membrane fluidity and membrane phospholipid conformation (Booyens and Thantsha, 2014). That was also confirmed by the significant changes in spectral intensity at the region 1,100–900 cm^−1^, which related to changes in cell wall polysaccharides or carbohydrates. In addition, the reduced tension of the amide I and amide II peaks of *L. plantarum* may originate from polysaccharides and cellular components covalently bound to peptidoglycan on the cell wall. Figure 6B also confirmed that this region is a key site for the effect of MDA on *L. plantarum*. For the significant change in *S. xylosus* in the amide II region, it has been established that certain antibiotics may reduce *S. aureus* polysaccharides, making the bacteria more susceptible to antibiotic treatment (Chew et al., 2022).

Furthermore, the reduced intensity of protein/amides I and II (1,700–1,500 cm^−1^) and carbohydrate bands (1,100–1,000 cm^−1^) also could reflect the highest degree of cell lysis (Jiang et al., 2004; Figure 6A). This suggested that the antimicrobial action of MDA is to target the peptidoglycan layer and alter membrane characteristics, which is consistent with the results of methicillin-resistant *S. aureus* by pyrogallol (Chew et al., 2022). Moreover, the key differences in the spectral features presented in Figure 6B may be caused by the strain-specific sensitivity of the strains to external stress.

## Conclusion

Overall, this study affirmed that MDA has obvious antibacterial activity on *S. xylosus* and *L. plantarum* with the MICs of 90 μg/ml and 180 μg/ml, respectively. In addition, the antibacterial mechanisms of MDA on *S. xylosus* and *L. plantarum* were associated with LDH, Ca^2+^ and Mg^2+^ leakage, cell membrane depolarization, and reduced intracellular ATP levels. The Combination of CLSM and FEGSEM observations affirmed that MDA disrupted the cell membrane of *S*. xylosus and *L*. plantarum. FT-IR studies further showed that MDA could affect the molecular composition of *S. xylosus* and *L. plantarum* cells. This study revealed that MDA, a marker of lipid oxidation, had an inhibitory effect on the dominant bacteria in dry-cured fish, providing a theoretical basis for further study on the interactions between lipid oxidation and microbial contamination. However, it is still necessary to further explore the antibacterial effects of the remaining RCSs and their specific metabolic effects on bacteria.

## Data availability statement

The original contributions presented in the study are included in the article/Supplementary material, further inquiries can be directed to the corresponding author.

## Author contributions

QZ: conceptualization, methodology, investigation, and writing-original draft. SJ: methodology and writing-review and editing. YiD: methodology and investigation. DL and YuD: writing-review and writing. XZ: conceptualization, supervision, and writing-review and editing. All authors contributed to the article and approved the submitted version.

## Funding

This work was supported financially by the National Natural Science Foundation of China (No. 31871869) and the National Key R&D Program of China (No. 2018YFD0901006).

## Conflict of interest

The authors declare that the research was conducted in the absence of any commercial or financial relationships that could be construed as a potential conflict of interest.

## Publisher’s note

All claims expressed in this article are solely those of the authors and do not necessarily represent those of their affiliated organizations, or those of the publisher, the editors and the reviewers. Any product that may be evaluated in this article, or claim that may be made by its manufacturer, is not guaranteed or endorsed by the publisher.
